# Controlled blood pressure elevation and limited fluid resuscitation in the treatment of multiple injuries in combination with shock

**DOI:** 10.12669/pjms.345.15465

**Published:** 2018

**Authors:** Yan Lu, Liping Liu, Jing Wang, Limin Cui

**Affiliations:** 1 Yan Lu, Binzhou People’s Hospital, Binzhou, Shandong, 256610, China; 2 Liping Liu, Binzhou Hospital of Traditional Chinese Medicine, Binzhou, Shandong, 256600, China; 3 Jing Wang, Binzhou People’s Hospital, Binzhou, Shandong, 256610, China; 4 Limin Cui, Binzhou People’s Hospital, Binzhou, Shandong, 256610, China

**Keywords:** Multiple injuries, Controlled blood pressure elevation, Limited fluid resuscitation

## Abstract

**Objective:**

To explore the effectiveness of controlled blood pressure elevation and limited fluid resuscitation in treating patients with multiple injuries in combination with shock in Intensive Care Unit (ICU).

**Methods:**

One hundred and sixty-four patients with multiple injuries in combination with shock who were admitted into the ICU of the hospital between June 2014 and November 2017 were selected and divided into an observation group and a control group using random number table, 82 each group. Controlled blood pressure elevation was given to both groups. Moreover, the control group was given conventional fluid resuscitation, while the observation group was given limited fluid resuscitation. The treatment effectiveness and complications were compared between the two groups.

**Results:**

The resuscitation time, post-resuscitation PT and post-resuscitation C-reactive protein level of the observation group were significantly lower than those of the control group (P<0.05). The post-resuscitation hemoglobin level of the observation group was significantly higher than that of the control group (P<0.05). The lactate clearance rate (LCR) of the observation group was (0.22±0.01) and (0.37±0.06) respectively three and six hours after fluid resuscitation, which was remarkably different with that of the control group ((0.27±0.03) and (0.51±0.08)) (P<0.05), but the difference became insignificant 24 h after fluid resuscitation (P>0.05). The observation group had significantly lower incidences of complications such as disseminated intravascular coagulation, respiratory distress syndrome and multiple organ dysfunction syndromes of the observation group and death rate than the control group, and the differences had statistical significance (P<0.05).

**Conclusion:**

Controlled blood pressure elevation in combination with limited fluid resuscitation is more effective than conventional fluid resuscitation in the treatment of patients with multiple injuries and shock in ICU as it can shorten recovery time, improve microcirculation perfusion and prognosis, and reduce related complications and fatality rate.

## INTRODUCTION

Multi-injury induced shock is a common emergency and severe disease. Active bleeding which is difficult to be controlled at first visit due to the severe condition of primary diseases may induce multi-organ functional disturbance and systemic inflammatory response syndrome, which can severely threaten the life safety of patients.^1^,^2^ Previously many researchers considered that positive fluid resuscitation was needed by patients with multi-injury induced hemorrhagic shock because it could accelerate effective circulation and ensure elevated and stable blood pressure and effective perfusion.^3^,^4^ However, it is also reported that the traditional resuscitation method may cause poor prognosis though it can rapidly recover effective circulating blood volume.^5^

People tend to have improved recognition on the pathogenesis of shock and damage control resuscitation in recent years. Controlled blood pressure elevation and limited fluid resuscitation method have been gradually popularized in the rescue of patients with multi-injury induced shock in Intensive Care Unit (ICU).^6^ Limited fluid resuscitation refers to keeping blood pressure of hemorrhagic shock patients with uncontrolled bleeding within a range which can satisfy the demand of organs on the lowest perfusion but will not cause excessive disturbances to the internal environment and compensatory mechanism by controlling the input quantity and speed of liquid.^7^,^8^ Fan et al. found that permissive hypertensive fluid resuscitation could effectively avoid side effects induced by early excessive resuscitation and maintain sufficient blood pressure to ensure blood supply of important organs.^9^ To further explore effective rescue measures, this study enrolled 164 patients with multi-injury induced shock and treated them with controlled blood pressure elevation in combination with limited fluid resuscitation and conventional fluid resuscitation, aiming to improve survival rate and promote recovery.

## METHODS

### General Data

This is a prospective study. One hundred and sixty-four patients with severe multi-injury induced shock who were admitted to the ICU of our hospital between June 2014 and November 2017 were selected. The inclusive criteria included being admitted to ICU because of severe multiple injuries, injury severity score higher than 16 points, with hemorrhagic shock, average arterial pressure lower than 65 mmHg or systolic pressure lower than 40 mmHg, and have undergone hemostatic treatment one or two hours after admission. Patients who died in 24 hours after admission to ICU or had craniocerebral trauma, severe cardiopulmonary and hepatic and renal dysfunction or severe hypertension were excluded. The patients were numbered according to the admission time and allocated to an observation group and a control group using random number table, 82 each group. This study satisfied medical ethical standards and has been approved by the ethics committee of Binzhou People’s Hospital. The included patients or their family members have signed informed consent.

### Therapeutic methods

The severity of the patients was evaluated immediately and scientifically after they went to hospital for treatment. The blood clot and secretions in the respiratory tract were cleaned, then measures such as sputum suction and oxygen uptake were used. Tracheal intubation or assisted mechanical ventilation was given to the patients according to their actual conditions. Next the arterial blood gas indexes of patients in the two groups were analyzed, the indexes such as electrocardiogram, blood pressure and blood oxygen were closely monitored, and more than two effective venous channels were established for fluid resuscitation. Balanced salt solution and colloidal solution were infused through veins. Skin and blood preparations were made after definite diagnosis. Emergency operation was performed as soon as possible after positive control of active hemorrhage. Complex surgery which lasted for at least 3 hours was arranged early. The room was disinfected for 10 minutes and ventilated for 10 minutes after every surgery. The frequency of going in and out surgical room was controlled. All the surgical articles were prepared before surgery. The number of medical staffs in the surgical room was controlled. The viewers were asked to keep a distance of 1 m with the patients. The nursing staffs who were familiar with surgical equipment coordinated with physicians to complete surgery, placed surgical instruments separately, and transferred surgical instruments following aseptic principle.

Patients in the control group were given conventional fluid resuscitation and controlled blood pressure elevation. Effective venous channels were established as soon as possible. Electrocardiogram was monitored all the day, and continuous oxygen inhalation treatment was performed. Plasma and equilibrium liquid were infused to recover effective blood volume when the vital signs were closely monitored. The mean arterial pressure (MAP) was kept between 60 mmHg and 80 mmHg to ensure the blood supply of important organs such as heart and brain.

Patients in the observation group were given controlled blood pressure elevation and limited fluid resuscitation. The changes of vital signs of the patients were closely monitored. 7.5% sodium chloride solution and plasma solution were infused. The MAP was controlled between 40 mmHg and 50 mmHg by controlling the infusion speed and volume of solution.

### Observation indicators

The recovery time and levels of hemoglobin (HGB), prothrombin time (PT) and C-reactive protein of patients in the two groups were recorded. The concentration of lactic acid in venous blood was detected using enzymic method before fluid resuscitation. The concentration of blood lactic acid was detected three hour and six hour after resuscitation. Lactate clearance rate (LCR) was calculated using a formula: LCR = (initial Lac value-Lac value at different time points after treatment)/initial Lac value×100 %.^10^ The incidences of complications such as disseminated intravascular coagulation, respiratory distress syndrome and multiple organ dysfunction syndrome and death rate were recorded.

### Statistical method

Data were statistically analyzed using SPSS ver. 21.0. Enumeration data were expressed as [n (%)] and processed by Chi-square test. Normally distributed measurement data were expressed as mean ± standard deviation (SD) and processed by independent sample t-test. P<0.05 indicated a significant difference.

## RESULTS

### Comparison of general data between the two groups

The observation group included 53 males and 29 females; they aged from 21 to 45 years (average 32.3±4.2 years); there were 37 cases of severe shock, 31 cases of moderate shock and 14 cases of mild shock. The control group included 57 males and 25 females; they aged from 22 to 47 years (average 33.6±4.2 years); there were 39 cases of severe shock, 33 moderate shocks and 10 mild shocks. The general data of the two groups had no remarkable differences (P>0.05); therefore the results were comparable.

### Comparison of treatment efficacy between the two groups

The recovery time, PT and C-reactive protein level of patients in the observation group were significantly lower than those in the control group (P<0.05). The HGB level of the observation group was much higher than that of the control group after resuscitation (P 0.05, Table-I).

**Table-I:** Recovery time, HGB, PT and C-reactive protein level between the two groups (mean±SD).

Group	Recovery time (min)	HGB (g/L)	PT (s)	C-reactive protein level (mg/L)
Observation group	89.7±25.2	102.5±13.0	10.1±1.2	101.7±12.3
Control group	193.5±38.7	84.6±8.3	16.9±2.4	132.4±20.6
t	13.508	6.913	14.187	8.286
P	0.000	0.006	0.000	0.000

### Comparison of lactate clearance rate between the two groups

The LCR of the observation group was significantly different with that of the control group three hour and six hour after fluid resuscitation (0.22±0.01 vs. 0.27±0.03; 0.37±0.06 vs. 0.51±0.08, Table-II).

**Table-II:** Lactate clearance rate between the two groups at different time points (mean±SD).

Group	Level of blood lactic acid before resuscitation (Lac, mmol/l)	LCR at different time points after resuscitation		

		3h	6h	24h
Observation group	5.73±1.29	0.22±0.01	0.37±0.06	0.77±0.04
Control group	5.94±1.61	0.27±0.03	0.51±0.08	0.76±0.04
t	0.352	6.491	8.593	0.271
P	0.163	0.008	0.000	0.179

### Comparison of incidences of complications and fatality rate between the two groups

The incidences of complications such as disseminated intravascular coagulation, respiratory distress syndrome and multiple organ dysfunction syndrome of the observation group were significantly lower than those of the control group. Two patients in the observation group and ten patients in the control group died. The death rate of the observation group was much lower than that of the control group, and the difference had statistical significance (P<0.05, Table-III).

**Table-III:** Incidences of complications and fatality rate between the two groups [n(%)].

Group	Disseminated intravascular coagulation	Respiratory distress syndrome	Multiple organ dysfunction syndrome	Fatality rate
Observation group	2(2.4%)	10(12.2%)	10(12.2%)	2(2.4%)
Control group	14(17.1%)	25(30.5%)	24(29.3%)	15(18.3%)
X^2^	4.322	6.868	5.107	4.183
P	0.039	0.006	0.027	0.041

## DISCUSSION

Patients with multi-injury induced shock in ICU usually have serious impairment of body functions. But massive blood loss can consume a large amount of blood coagulation factor. Therefore many patients have had cell metabolism, abnormal coagulation function and insufficient tissue perfusion before going to hospitals.^11^,^12^ It is widely believed that the first step to rescue patients with multi-injury shock in ICU is to establish venous channels and timely infuse fluid to promote recovery of effective circulation and stablize blood pressure.^13^ Previously fluid resuscitation took crystoloid solution as dilatation fluid and rapid perfusion of crystoloid solution in a short time could dilute blood coagulation factors in blood, damage functions of platelet, and further aggravate coagulation disorders. Therefore someone put forward limited fluid resuscitation treatment.^14^ Rapid infusion should be avoided and blood pressure may not be intentionally recovered to the normal range before active hemorrhage of patients with multi-injury induced shock in ICU has been effectively controlled;^15^ full resuscitation can be performed after the basic demands of body are maintained after surgical hemostasis. The quantitative standard of limited fluid resuscitation is controlled blood pressure elevation. The fluid infusion amount of different patients is different because of the individual differences. Therefore controlling mean arterial pressure (MAP) between 40 mmHg and 50 mmHg can increase the survival rate of patients in 72 h, which is positive to prevent early death.

The results of this study suggested that the recovery time, PT, C-reactive protein level and HGB of the observation group were (89.7±25.2) minutes, (10.1±1.2) s, (101.7±12.3) ng/L and (102.5±13.0) g/L respectively, which were significantly different with those of the control group; the finding was consistent with the study of Xu.^16^ For patients with multi-injury induced shock, excessive fluid infusion in ICU will dilute blood and affect coagulation function; C-reactive protein and PT can directly reflect the coagulation function and tissue infection and injury of patients, which have important values in the treatment and prognosis of patients.^17^

Concentration of arterial blood lactate is one of the highly sensitive indicators which can reflect tissue hypoxia.^18^ The level of blood lactate may increase because of the large amount of adenosine triphosphate consumed by increased oxygen consumption and decompensated tissue hypoxia. A high level of lactate can indicate increased fatality rate. Recovery of concentration of blood lactate in the first 24 hour after resuscitation is extremely crucial. LCR can better reflect the prognosis of patients than value of blood lactate.^19^ The present study found that blood lactic acid was not effectively cleaned and microcirculation and oxygen metabolism was not improved in the early stage of resuscitation in the observation group; but twenty-four hours after resuscitation, blood lactic acid was effectively eliminated, microcirculation perfusion and tissue oxygen metabolism were improved in both groups. To maintain the perfusion pressure below the control group, the observation group was given a small amount of fluid, which could not obviously improve early microcirculation perfusion; therefore the LCR decreased. But twenty-four hours after treatment, patients in both group received surgical hemostasis besides fluid resuscitation. Hence the insignificant difference of LCR between the two groups might be correlated to the timely surgical hemostasis and sufficient postoperative fluid perfusion. It was concluded that the patients who were treated using the two schemes had significantly improved tissue perfusion and tissue oxygen metabolism 24 hours after treatment. The results of this study suggested that the incidence of complications and fatality rate of the observation group were significantly lower than those of the control group, which was consistent with the finding of Li and He^20^ i.e., the incidence of multiple organ dysfunction syndrome, incidence of disseminated intravascular coagulation and fatality rate of the test group (7.35%, 12.45% and 2.53%) were significantly lower than those of the control group. Limited fluid resuscitation will firstly recover blood flow perfusion, relieve acidosis, prevent excessive disturbance to body environment and compensatory mechanism; and then the tissue perfusion and oxygen supply will be improved; as a result, the incidence of relevant complications can be reduced. Therefore we recommend that active hemorrhage which happens in the early stage in the process of limited fluid resuscitation should be treated as soon as possible to prevent disturbance to the compensatory mechanism of hemorrhage.

### Limitations of this study

The research time was short, and moreover it was a single-center study with a small sample size; hence some errors were inevitable. Therefore randomized controlled trials with a large sample size are needed to further verify the accuracy of those indicators.

## CONCLUSION

The clinical efficacy of controlled blood pressure elevation and limited fluid resuscitation is superior to conventional fluid in the treatment of patients with multi-injury induced shock in ICU. Controlled blood pressure elevation in combination with limited fluid resuscitation can be popularized because it can effectively shorten resuscitation time, reduce incidences of related complications and death rate and improve prognosis.

### Authors’ Contribution

**YL & LMC:** Study design, data collection and analysis.

**YL, LPL & JW:** Manuscript preparation, drafting and revising.

**LMC:** Review and final approval of manuscript.
